# Beneficial Effects of Tannic Acid on the Quality of Bacterial Communities Present in High-Moisture Mulberry Leaf and Stylo Silage

**DOI:** 10.3389/fmicb.2020.586412

**Published:** 2020-11-02

**Authors:** Cheng Wang, Ruiqi Pian, Xiaoyang Chen, Hongjian Lv, Wei Zhou, Qing Zhang

**Affiliations:** ^1^ College of Forestry and Landscape Architecture, Guangdong Province Research Center of Woody Forage Engineering Technology, Guangdong Research and Development Center of Modern Agriculture (Woody Forage) Industrial Technology, South China Agricultural University, Guangzhou, China; ^2^ Guangdong Key Laboratory for Innovative Development and Utilization of Forest Plant Germplasm, State Key Laboratory for Conservation and Utilization of Subtropical Agro-bioresources, South China Agricultural University, Guangzhou, China

**Keywords:** bacterial community, mulberry leaf, silage quality, *Stylosanthes guianensis*, tannic acid

## Abstract

Tannic acid (TA), a type of polyphenol, is widely distributed in plants, especially in legumes. Not only does it possess antimicrobial properties, but it also has the ability to bind with proteins. The fermentation parameters, nitrogen fractions, antioxidant capacity, and bacterial communities present in mulberry leaves and stylo (*Stylosanthes guianensis*) ensiled with or without 1 and 2% TA per kilogram of fresh matter (FM) were investigated after 75 days’ fermentation. The results showed that 1 and 2% TA both significantly decreased the butyric acid content (4.39 and 7.83 g/kg dry matter (DM), respectively) to an undetectable level in both mulberry leaf and stylo silage. In addition, 2% TA significantly increased the contents of lactate (24.0–39.0 and 8.50–32.3 g/kg DM), acetate (18.0–74.5 and 9.07–53.3 g/kg DM), and the antioxidant capacity of both mulberry leaf and stylo silage, respectively. With the addition of 1 and 2% TA, the pH values (5.55–5.04 and 4.87, respectively) and ammonia-N (NH_3_-N) content (85.5–27.5 and 16.9 g/kg total nitrogen (TN), respectively) were all significantly decreased in stylo silage. In addition, TA increased the relative abundance of *Weissella*, *Acinetobacter*, and *Kosakonia* spp. and decreased that of undesirable *Clostridium* spp. TA can thus be used to improve the silage quality of both mulberry leaf and stylo silage, with 2% TA being the better concentration of additive to use.

## Introduction

Ensiling is a more appropriate technology for preserving fresh forage than traditional hay-making, as it not only reduces nutritional losses but also reduces production costs, especially in the rainy season (Liu et al., 2011). Moisture is a crucial factor for silage quality, because a raw material with a high dry matter (DM) content can produce weak fermentation due to lack of moisture for active fermentation or even aerobic deterioration, caused mainly by *Clostridium* (Han et al., 2014). However, satisfactory fermentation quality is difficult to obtain due to proteolysis and clostridial fermentation during the ensiling process. It is well-known that proteins are hydrolyzed to free amino acids and peptides, due mainly to plant proteases in the early stages of ensiling, and then these intermediates are further deaminized to ammonia and other end products as a result of microbial activity. When ingested by ruminants, such nonprotein nitrogen cannot be fully utilized by rumen microorganisms, consequently resulting in nitrogen losses *via* animal excreta and environmental pollution. Therefore, it is necessary to discover strategies for preventing or decelerating protein degradation during ensiling in order to maintain the nitrogen (N)-utilization efficiency of ruminants and minimize *N* emissions to the environment (Anuraga et al., 2018).

Tannic acid (TA), a type of water-soluble plant polyphenol, has numerous pharmacological applications. It is low cost and easy to obtain because it is widely distributed in both legumes and nonlegumes (Silanikove et al., 2001) and is also used as an antioxidant in the food and beverage industry. Furthermore, TA can bind to proteins and thus inhibit their being broken down by proteases in both ensiled silos and in the rumen. Numerous studies have shown that TA can reduce proteolysis during ensiling (Guo et al., 2007; Li et al., 2018; He et al., 2020). Guo et al. (2007) and Li et al. (2018) found that the addition of TA reduced proteolysis by decreasing the activity of acid proteinase, carboxypeptidase, and aminopeptidase in alfalfa silage. Apart from protecting proteins from degradation, TA also showed positive effects on improving the productivity of livestock (Mueller, 2006) and reducing ruminal methane emissions (Tiemann et al., 2008). Fraser et al. (2000) reported that the DM intakes of cows were similar to each other but that the milk and protein yields were higher when the animals were fed silage prepared from tannin-containing plants.

Additionally, TA has been reported to possess antimicrobial activity toward an extensive range of microbes (Mueller, 2006; Daglia, 2012). However, to our knowledge, few studies have been conducted on the effects of TA on the microbial communities, especially those of *Clostridium* spp., in silage. It is still unknown whether the proteolysis reduction caused by the addition of TA is related to its antibacterial action.

The white mulberry (*Morus alba*) comprises 10–13 species and more than 1,000 cultivars widely distributed throughout Asia, Europe, Africa, and North America (Nepal and Ferguson, 2012), which are well-known for their economic and medicinal value (Wang et al., 2019a). Stylo (*Stylosanthes guianensis* Sw.), a flowering legume commonly known as pencilflower, grows mainly in tropical and subtropical regions and is an important feed source for ruminants, with high yields and high nutrient levels (He et al., 2019b). However, few studies have focused on the effects of TA on the quality of silage fermentation in these two plant species. Therefore, mulberry leaves and stylo were ensiled with and without the addition of 1 or 2% TA solution for a period of 75 days’ fermentation at room temperature, following the bacterial communities, nitrogen fractions, antioxidant capacities, and fermentation quality of the two types of silage were analyzed.

## Materials and Methods

### Silage Preparation

Mulberry (Yuesang 11) and stylo (CIAT 184) were planted on adjacent experimental farms at the South China Agricultural University (23°19' N; 113°34' E, Guangdong, China) and cultivated without herbicides or fertilizers. We collected fresh mulberry leaves and stylo on January 26, 2019, and the different harvested materials were chopped into 20 mm lengths using a handy cutter (Model 9ZP-3.6, Kaiyue Machinery Company, China). Following homogenization, the two raw materials were collected in triplicate samples and were used for determining chemical composition and microbial populations. The 1 and 2% TA solutions were prepared by dissolving 1.5 and 3.0 g accurately weighed quantities of the acid (CAS: 5995-86-8; purity ≥98%; Macklin, China) in 10 ml of sterile distilled water. Then a given amount of mulberry leaves and stylo (about 150 g) were treated with (i) 10 ml of sterile distilled water (CK), (ii) 1% TA, and (iii) 2% TA, respectively. The materials were then immediately packed into lab-level silo bags (20 × 30 cm; Dongguan Bojia Packaging, China), which were sealed with a vacuum sealer (Lvye DZ280, Yijian Packaging Machinery Co. Ltd., China). In total, 24 bags (2 forages × 3 treatments × 4 replicates) were individually prepared and kept at room temperature (25–30°C). After 75 days’ fermentation, four bags from each treatment were randomly sampled to analyze their fermentation characteristics, protein fractions, tannin compositions, and bacterial communities.

### Analyses of Microbial Composition, Fermentation Parameters, Protein Fractions, Tannin Compositions, and Antioxidant Capacity

The experimental procedures described in this section were similar to those previously reported (He et al., 2019b). Briefly, 20 g silage samples were homogenized in 180 ml of sterilized saline for 30 min in an orbital shaker, and the supernatants were then serially diluted from 10^−1^ to 10^−6^. Lactic acid bacteria (LAB) and coliform bacteria were separately cultivated on Man Rogosa Sharpe (MRS) agar and Violet Red Bile agar for 48 h in aerobic incubator. Yeast and mold counts were estimated using Rose Bengal agar following incubation at 28°C for 72–120 h. According to the method described by Wang et al. (2019a), further 20 g samples were mixed with 180 ml distilled water and stored at 4°C for 18 h and filtered to determine their fermentation characteristics. The pH was measured directly with a pH meter (PHS-3C, INESA Scientific Instrument Co., Ltd., Shanghai, China), ammonia-N (NH_3_-N) content was determined by the phenol-hypochlorite colorimetric method, and organic acids (lactic acid, acetic acid, propionic acid, and butyric acid) were analyzed by high-performance liquid chromatography [HPLC; column, Shodex RSpak KC-811S-DVB gel C (8.0 mm × 30 cm; Shimadzu, Tokyo, Japan); oven temperature, 50°C; mobile phase, 3 mmol/L HClO_4_; flow rate, 1.0 ml/min; injection volume, 5 μl; and detector, SPD-M10AVP; Zhang et al., 2016]. The remaining samples were oven-dried and ground for chemical analysis. Protein fractions (crude protein, CP and true protein, TP) were analyzed using a Kjeldahl nitrogen analyzer (Kjeltec 2300 Auto Analyzer, FOSS Analytical AB, Hoganas, Sweden) according to the methods of the Association of Official Analytical Chemists (AOAC, 1990). Fiber fractions (neutral detergent fiber, NDF and acid detergent fiber, ADF) were analyzed by the method of Van Soest et al. (1991) without the use of heat-stable amylase and sodium sulfite. Water-soluble carbohydrates (WSCs) were measured by the anthrone method (Murphy, 1958).

Total phenols, simple phenols (SPs), and hydrolysable tannins (HTs) were measured by the Folin-Ciocalteu colorimetric method (Makkar, 2010). According to He et al. (2020), radical 2,2-diphenyl-1-picrylhy-drazyl (DPPH) scavenging activity, radical 2,2-azinobis-3-ethylben-zothiazoline-6-sulfonic acid diammonium salt radical cation (ABTS) scavenging activity, and ferric-reducing antioxidant power (FRAP) were each measured and combined to represent the total antioxidant capacity. Trolox was used to establish a standard line, and the scavenging activity and reducing power were expressed as milligrams of trolox equivalents per gram of DM (mg TE/g DM).

### Bacterial Community Sequencing Analysis

Total metagenomic DNA was extracted with a DNA Kit (Omega Biotek, Norcross, GA, United States) following the manufacturers’ instructions. PCRs were conducted in a 50 μl mixture, including 5 μl of 2.5 mM dNTPs, 5 μl of 10 × KOD Buffer, 1.5 μl of each primer (5 μM), 1 μl of KOD polymerase, and 100 ng of template DNA. The V3–V4 regions of 16S rDNA were amplified using the primers (341F: CCTACGGGNGGCWGCAG; 806R: GGACTACHVGGGTATCTAAT). The PCRs were conducted in the same mixture system and using the same reaction procedures as detailed by He et al. (2019c), and were then purified and quantified. Subsequently, the purified PCR products were sequenced on an Illumina HiSeq 2500 Sequencing System (Illumina, Inc., San Diego, CA, United States), and the raw sequences were analyzed as according to the procedures described by Wang et al. (2018). Finally, the effective tags were clustered into operational taxonomic units (OTUs) at 97% similarity using the UPARSE pipeline. Taxonomy assignment of representative sequences was performed using the Ribosome Database Project (RDP) classifier (Version 2.2). The α-diversity was calculated in the QIIME bioinformatic pipeline.^1^ Principal component analysis (PCA) was also used to analyze β-diversity. The relative abundances of different bacterial communities at the genus level were also analyzed. The sequences were deposited in the sequence read archive (SRA) with the accession number SRP220981.

### Statistical Analysis

The IBM SPSS 20.0 for Windows statistical software package was used to analyze the effects of TA addition on the fermentation characteristics of mulberry leaves and stylo silage. The effects were evaluated using one-way ANOVA, with Duncan’s multiple range tests. Statistical significance was determined at the <0.05 level. An online platform^2^ was used to analyze the sequencing data of the bacterial community.

## Results

### Characteristics of Fresh Material Before Ensiling

The chemical composition and microbial population of fresh mulberry leaves and stylo prior to ensiling are listed in Table 1. The DM contents of the two materials were 278 and 249 g/kg, respectively. The CP contents of mulberry leaves and stylo were 196 and 120 g/kg DM, respectively, and their WSC contents were 126 and 18.3 g/kg DM, respectively. The LAB counts were 4.11 and 4.62 log_10_ colony forming units (CFUs)/g fresh matter (FM), while the coliform bacteria counts were 5.4 and 6.38 log_10_ CFU/g FM, respectively.

**Table 1:** Chemical composition and microbial population of fresh mulberry leaves and stylo prior to ensiling (±*SD*, *n* = 3).

Items	Mulberry leaves	Stylo
Dry matter (g/kg FM)	278 ± 5.4	249 ± 3.0
Crude protein (g/kg DM)	196 ± 0.82	120 ± 2.5
True protein (g/kg TN)	885 ± 9.3	874 ± 35
Non-protein nitrogen (g/kg TN)	115 ± 9.3	126 ± 35
Neutral detergent fiber (g/kg DM)	199 ± 5.1	441 ± 15.0
Acid detergent fiber (g/kg DM)	107 ± 4.3	331 ± 12.0
Water-soluble carbohydrate (g/kg DM)	126 ± 3.6	18.3 ± 1.6
Lactic acid bacteria (log_10_ CFU/g FM)	4.11 ± 0.13	4.62 ± 0.15
Yeasts (log_10_ CFU/g FM)	<3.00	4.76 ± 0.25
Molds (log_10_ CFU/g FM)	<3.00	3.53 ± 0.21
Coliform bacteria (log_10_ CFU/g FM)	5.40 ± 0.26	6.38 ± 0.11

FM, fresh matter; DM, dry matter; TN, total N; and CFU, colony forming units.

### Fermentation Quality, Microbial Populations, and Chemical Characteristics of Both Mulberry Leaf and Stylo Silage

The fermentation quality, microbial populations, and chemical characteristics of both mulberry leaf and stylo silage after 75 days’ ensiling are listed in Tables 2–4. The pH values were in the ranges 6.51–6.89 and 4.89–5.55 for both mulberry leaf and stylo silage, respectively. TA decreased (*p* < 0.01) the pH value and increased (*p* < 0.05) the lactic acid and acetic acid content in stylo silage. In addition, TA decreased the butyric acid content in both mulberry leaves and stylo silage. Propionic acid was not detected in this study. The LAB count tended to decrease with TA addition for both mulberry leaf (*p* > 0.05) and stylo silage (*p* < 0.05). The number of coliform bacteria was high (6.08–6.42 log_10_ CFU/g FM) in mulberry leaf silage, while they were not detected in stylo silage. The numbers of molds and yeasts were below detection levels in both types of silage. Stylo silage with 2% TA had significantly lower (*p* < 0.05) proportions of nonprotein-N (NPN) and NH_3_-N and a higher (*p* < 0.05) proportion of total nitrogen (TN), while only CP and free amino acid-N (FAA-N) had reduced proportions (*p* > 0.05). Mulberry leaf silage had a relatively high proportion of NH_3_-N, and 1% TA additive further increased (*p* < 0.05) the NH_3_-N proportion. As shown in Table 4, TA also deceased (*p* < 0.01) the concentrations of total phenols, SP, and HT than the control in the two types of silage. In addition, the antioxidant capacity of mulberry leaf and stylo silage improved remarkably (*p* < 0.01) with TA addition.

**Table 2:** Organic acid contents, pH, and microbial population of ensiled mulberry leaves and stylo.

Items	Mulberry leaves					Stylo				
	CK	T1	T2	SEM	*p* value	CK	T1	T2	SEM	*p* value
Dry matter (g/kg FM)	270	266	285	2.53	0.013	258	252	261	6.08	0.870
pH	6.51	6.89	6.74	0.057	0.005	5.55	5.04	4.87	0.094	<0.001
Lactic acid (g/kg DM)	24.0	26.1	39.0	2.43	0.006	8.50	15.0	32.3	5.32	<0.001
Acetic acid (g/kg DM)	18.0	40.9	74.5	8.14	0.002	9.07	16.5	53.3	8.90	<0.001
Propionic acid (g/kg DM)	ND	ND	ND	—	—	ND	ND	ND	—	—
Butyric acid (g/kg DM)	4.39	ND	ND	0.838	0.027	7.83	ND	ND	1.16	<0.001
Lactic acid bacteria (log_10_ CFU/g FM)	7.61	6.89	6.74	0.069	0.287	5.89	3.71	3.85	0.833	0.001
Molds (log_10_ CFU/g FM)	<2.00	<2.00	<2.00	—	—	<2.00	<2.00	<2.00	—	—
Yeasts (log_10_ CFU/g FM)	<2.00	<2.00	<2.00	—	—	<2.00	<2.00	<2.00	—	—
Coliform bacteria (log_10_ CFU/g FM)	6.42	6.08	6.25	0.048	0.002	<2.00	<2.00	<2.00	—	—

FM, fresh matter; DM, dry matter; CFU, colony forming units; ND, not detected; SEM, standard error of means; CK, control; T1, 1% FM TA added; and T2, 2% FM TA added.

**Table 3:** Protein fractions of ensiled mulberry leaves and stylo.

Items	Mulberry leaves					Stylo				
	CK	T1	T2	SEM	*p* value	CK	T1	T2	SEM	*p* value
Crude protein (g/kg DM)	214	203	195	2.52	<0.001	121	118	119	0.763	0.369
True protein (g/kg TN)	499	536	484	8.59	0.019	440	573	650	26.5	<0.001
Nonprotein-N (g/kg TN)	501	464	516	8.59	0.019	560	427	350	26.5	<0.001
Free amino acid (g/kg TN)	16.1	13.0	12.6	0.71	0.303	29.5	24.2	21.3	1.94	0.226
Ammonia-N (g/kg TN)	44.3	53.6	46.1	2.39	0.001	85.6	27.5	16.9	9.44	<0.001

DM, dry matter; TN, total N; SEM, standard error of means; CK, control; T1, 1% FM TA added; and T2, 2% FM TA added.

**Table 4:** Tannin contents and antioxidant activities of ensiled mulberry leaves and stylo.

Items	Mulberry leaves					Stylo				
	CK	T1	T2	SEM	*p* value	CK	T1	T2	SEM	*p* value
Total phenol (g/kg DM)	3.28	4.22	7.37	0.543	<0.001	1.91	8.64	18.5	2.09	<0.001
Simple phenol (g/kg DM)	1.35	1.73	2.37	0.241	<0.001	1.15	1.30	2.74	0.225	<0.001
Hydrolysable tannin (g/kg DM)	1.93	2.49	5.00	0.420	<0.001	0.764	7.34	15.8	1.88	<0.001
DPPH	8.25	14.2	16.9	1.25	0.002	8.95	28.2	96.1	11.5	<0.001
ABTS	3.13	4.73	8.58	0.794	0.002	4.75	16.1	52.2	6.18	<0.001
FRAP	4.95	5.95	14.5	1.32	<0.001	4.59	11.5	39.2	4.57	<0.001

DPPH (mg of trolox equivalent/g of DM), free radical DPPH scavenging activity; ABTS (mg of trolox equivalent/g of DM), radical ABTS scavenging activity; and FRAP (mg of trolox equivalent/g of DM), ferric-reducing antioxidant power. DM, dry matter; TN, total N; SEM, standard error of means; CK, control; T1, 1% FM TA added; and T2, 2% FM TA added.

### Bacterial Diversity of Both Mulberry Leaf and Stylo Silage

**Figure 1:**
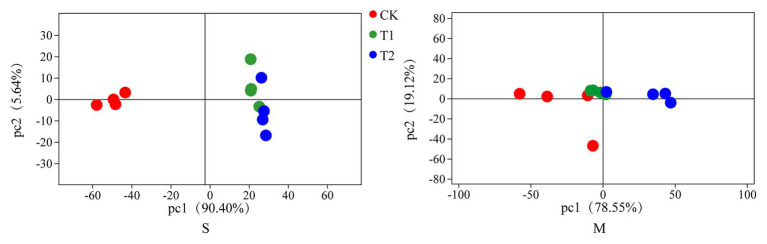
Principal component analysis (PCA) of bacterial communities in mulberry leaf (M) and stylo (S) silage with the addition of tannic acid (TA; CK, blank control; T1, 1% FM TA; and T2, 2% FM TA).

**Figure 2:**
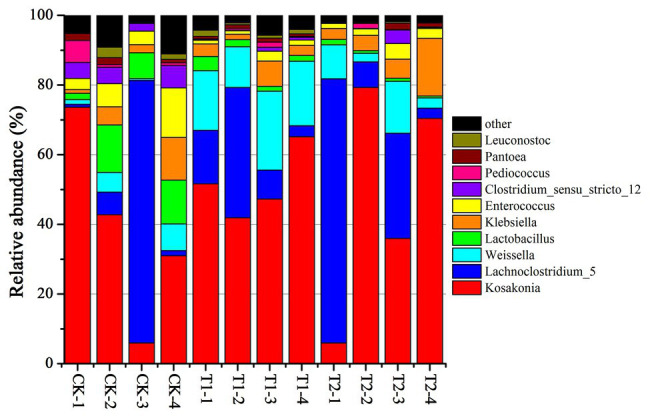
Relative abundance of bacterial communities at the genus level in mulberry leaf silage with the addition of TA (CK, blank control; T1, 1% FM TA; and T2, 2% FM TA).

**Figure 3:**
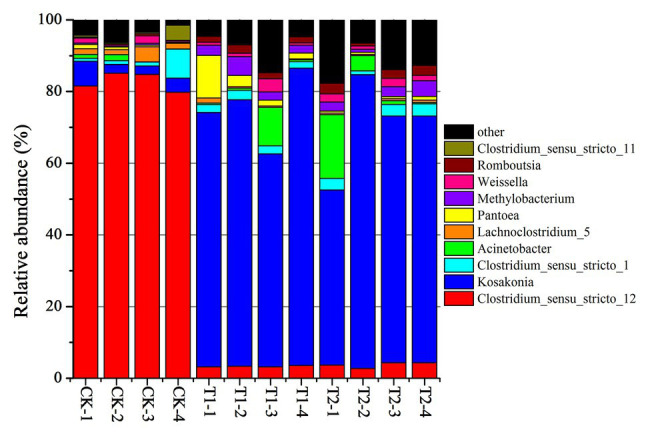
Relative abundance of bacterial communities at the genus level in stylo silage with the addition of TA (CK, blank control; T1, 1% FM TA; and T2, 2% FM TA).

The bacterial diversities of both mulberry leaf and stylo silage are listed in Table 5. Overall, the coverage values of all treatments were approximate 0.99, revealing that most of the bacteria were detected. For mulberry leaf silage, fewer OTUs but more Sobs, Chao1, and Ace were identified in the TA-treated groups compared to the control groups. By contrast, TA application led to an increase in Sobs, Chao1, and Shannon, as well as an increase in OTUs in stylo silage. PCA was applied, as shown in Figure 1. In this study, principal coordinates 1 (Pc1) and 2 (Pc2) were 78.55 and 19.12% of the total variance in mulberry leaf silage and 90.4 and 5.64% of the total variance in stylo silage, respectively. A clear separation was observed between the control groups and the TA-treated groups for both mulberry leaf and stylo silage. Moreover, a higher extent of separation was formed in stylo silage than in mulberry leaf silage. The relative abundances of bacterial communities at the genus level in both mulberry leaf and stylo silage are shown in Figures 2, 3. Overall, the relative abundances of the bacterial communities at the genus level were wide variability in both mulberry leaf and stylo silage, and were significantly changed with TA addition. In mulberry leaves silage, *Lachnoclostridium* (49.5%), *Kosakonia* (22.3%), *Lactobacillus* (7.97%), and *Enterococcus* (5.08%) were the dominant genera in the CK group, while the TA-treated group was dominated by *Lachnoclostridium* (18.1 and 32.7%), *Kosakonia* (51.0 and 45.8%), *Weissiella* (16.8 and 7.54%), and *Klebsiella* (3.47 and 6.13%). In stylo silage, the top three genera of the CK groups were *Clostridium* (87.1%), *Kosakonia* (3.86%), and *Lachnoclostridium* (2.82%), while *Kosakonia* (72.5 and 64.7%), *Clostridium* (5.53 and 6.71%), *Pantoea* (4.30% in the 1% TA-treated group), *Acinetobacter* (6.32% in the 2% TA-treated group), and *Methylobacterium* (2.90 and 2.61%) were the most dominated genera in the TA-treated groups.

**Table 5:** Alpha diversity of bacterial community for mulberry leaves and stylo silage.

Item	Treatment	OTUs	Sobs	Chao1	Ace	Shannon	Coverage
Mulberry leaves	CK	159	403	640	656	2.32	0.99
	T1	124	465	703	705	2.25	0.99
	T2	117	431	651	658	2.15	0.99
Stylo	CK	480	542	899	853	2.59	0.99
	T1	520	553	819	818	3.22	0.99
	T2	590	557	808	790	3.03	0.99

CK, control; T1, 1% FM TA added; and T2, 2% FM TA added.

## Discussion

### Characteristics of Fresh Material Prior to Ensiling

The CP content of mulberry leaves was comparable with that reported in our previous study (Wang et al., 2019a) but lower than the results reported in other studies (Iqbal et al., 2012), while the CP content of stylo was slightly lower than that determined by Wang et al. (2019b). Such variations might have resulted from the different effects of plant variety, climate, fertilization, and time of harvest on the chemical composition of the forage (Zhang et al., 2016). The relatively high CP content and low fiber content (199 and 107 g/kg DM for NDF and ADF, respectively) showed that mulberry leaves can be developed as a high-quality protein feed for livestock.

The WSC content is a crucial factor dictating silage fermentation quality. In this study, the WSC content of mulberry leaves was much higher than 60–70 g/kg DM, the theoretical requirement for obtaining well-preserved silage (Smith, 1962). By contrast, the high fiber content (441 and 331 g/kg DM for NDF, ADF, respectively) and low WSC content of stylo were not beneficial to ensilage and digestion. Furthermore, the epiphytic LAB in fresh forage is considered to be a significant factor for silage fermentation, at least 5 log_10_ CFU/g FM LAB during ensiling being necessary to produce well-preserved silage (Cai et al., 1998). The LAB counts of mulberry leaves and stylo were relatively low, while the counts for undesirable coliform bacteria were relatively high, which indicated that silage additives were necessary to ensure silage of sufficient quality for feeding to livestock. Anuraga et al. (2018) reported that tannins had the ability to limit extensive proteolysis. Therefore, TA improve the fermentative quality of silage may because the less proteolysis occurred in ensiling process. Thus, addition of TA might help to reduce protein loss and improve the fermentation quality during mulberry leaf and stylo ensiling.

### Fermentation Quality and Microbial Population of Both Mulberry Leaf and Stylo Silage

The ultimate intention of ensiling is to preserve biomass and reduce nutrient loss (He et al., 2019a). The pH value is an important parameter for estimating the quality of silage fermentation, where a pH ≤ 4.2 is necessary to produce well-preserved silage (McDonald et al., 1991). In the present study, the pH value was much higher than 4.2. Wang et al. (2019a) also reported that the pH value of mulberry leaf silage remained at 5.0 after 60 days of ensiling. This behavior might be ascribed to a high buffering capacity and extensive proteolysis, which generated ammonia and blocked a decline in pH (Wang et al., 2019b). It was consistent with our previous study; He et al. (2019b) reported that gallic acid decreased pH value (6.51 vs. 5.98 and 5.55 vs. 4.57 in mulberry leaves and style silage, respectively). Abundant coliform bacteria were present in mulberry leaf silage, as most spoilage microbes are usually inhibited at a pH < 4.5 during ensiling (Kung, 2009; Li et al., 2017). And the numbers of molds and yeasts were below 2.00 log_10_ CFU/g FM in all samples of silage. In this study, TA decreased the pH value of stylo silage, in line with the increase in lactic acid and acetic acid in the TA-treated groups. These results showed that TA might enhance the quality of silage fermentation. The growth of undesirable acetic acid-producing bacteria might metabolize lactic acid to acetic acid under sugar-deficient conditions (Parvin and Nishino, 2010). Seglar (2003) reported that acetic acid was an inhibitor of the growth of spoilage microorganisms and increased the aerobic stability. In addition, propionic acid was not detected, and butyric acid was detected only in the control group. Butyric acid is undesirable in silage because of the nutritional damage caused by secondary fermentation as a result of clostridial activity (McDonald et al., 1991). Hence, the decrease in butyric acid with addition of TA showed that the growth of harmful microorganisms such as *Clostridium* was inhibited during ensiling. This suggests that the use of TA as a silage additive can improve the quality of fermentation.

### Nitrogen Fractions of Both Mulberry Leaf and Stylo Silage

In principle, protein degradation is inevitable in the ensiling process. Proteolysis transforms TP to NPN such as FAA-N and NH_3_-N (Wang et al., 2019b). As a result, the silage produces an excessive quantity of rumen-degradable protein, which cannot be utilized by rumen microorganisms. The nitrogen loss resulting from animal excreta causes environmental pollution. Therefore, it is necessary to develop strategies to prevent or decelerate such protein degradation during the ensiling process in order to preserve forage nutrition and minimize *N* emissions to the environment (Anuraga et al., 2018). In the present study, inconsistent changes in protein fractions were shown in both mulberry leaf and stylo silage. Tabacco et al. (2006) reported that forage species, maturity sampling stage, moisture content, and rate of pH decline all affected silage proteolysis. The 2% TA decreased the NPN and NH_3_-N proportions, while increased the TN proportions of stylo silage. And CP and FAA-N proportions had no significant change. As He et al. (2019b) reported, NH_3_-N (0.71 vs. 0.19%, 1.46 vs. 0.29% TN) content also decreased in mulberry leaf and stylo silage with gallic acid addition. Thus, it is believed that the nutritional value of protein remains high in stylo silage.

These results were consistent with those of Ding et al. (2013), who reported that the ratios of NPN and NH_3_-N to TN both decreased in alfalfa silage with TA. It might be that the addition of TA resulted in a decline in deamination of amino acids or peptides in the silage, reducing the generation of NH_3_-N. Numerous studies have also shown that TA has the ability to inhibit plant peptidase activity and microbial proteinases (Albrecht and Muck, 1991) as well as form complexes with forage proteins (Deaville et al., 2010) during ensiling. Moreover, these tannin-protein complexes can exist over a wide pH range, from 3.5 to 7.5 in an acidic environment (Barry and McNabb, 1999).

However, the mulberry leaf silage in our study had a relatively high proportion of NH_3_-N, and 1% TA additive further increased that proportion. This was similar to the results in our previous study, where we reported that a high NH_3_-N content (0.5–1.6 g/kg DM) existed in mulberry leaves after 60 days’ ensiling (Wang et al., 2019b).

Dipeptidase, carboxypeptidase, and tripeptidyl-peptidase were the principal exopeptidases responsible for proteolysis during the process of alfalfa ensiling (Tao et al., 2012). He et al. (2019b) reported that their optimal pH values were 8.8, 5.0, and 7.0, respectively. Therefore, the increased proteolysis activities in mulberry leaf silage might be due to the higher protease activities because the TA-treated groups provided a favorable pH environment for the optimal activities of these proteases.

### Tannin Content and Antioxidant Capacity of Both Mulberry Leaf and Stylo Silage

The two types of silage treated with TA both had higher concentrations of total phenols, SP, and HT than the controls. In addition, the antioxidant capacities of both mulberry leaf and stylo silage improved remarkably with TA addition. Interestingly, the TA-treated groups had a greater HT content in stylo silage (8.64–18.5 g/kg DM) than mulberry leaf silage (2.49–5.00 g/kg DM). Correspondingly, the DPPH and ABTS radical scavenging activity and the FRAP of the TA-treated stylo silage were both much higher than that of the TA-treated mulberry leaf silage, which showed that the antioxidant capacity was direct proportional to HT content. Tumer et al. (2015) reported that high antioxidant activity in plant material is attributable mainly to the presence of polyphenols and quercetin. Anuraga et al. (2018) reported that HT had a greater ability to limit proteolysis in comparison with condensed tannins, which was related to their greater protein precipitation capacity. This is due to the particular chemical structure of HT, which contain carbohydrate as the central core, with abundant hydroxyl group substituents. This can explain the more ideal nitrogen fraction of stylo silage as compared with mulberry leaf silage. However, HT may be toxic to ruminants when used in excessive amounts (Reed, 1995). As Kumar and Vaithiyanathan (1990) reported, sheep that ingested 0.9 g HT/kg body weight showed signs of toxic poisoning after 15 days. Therefore, such negative effects on livestock feed must be avoided by adjusting the amount of feed per day when TA is used as a silage additive.

### Bacterial Diversity of Both Mulberry Leaf and Stylo Silage

Bacteria play a crucial role in the ensiling process. Therefore, monitoring the changes in different bacterial communities during fermentation should yield a good understanding of the proteolytic process (He et al., 2019a). The coverage values of all treatments were approximate 0.99, indicating that the data from sampling were sufficiently large to represent all of the bacterial communities present in the different samples of silage. The numbers of observed OTUs (Sobs), richness (Chao1 and Ace indexes), and diversity (Shannon index) were used to estimate the alpha diversity of the bacteria in each sample. Moreover, the differences in these indexes of stylo silage with the addition of TA were greater than those of mulberry leaves silage. For the latter, fewer OTUs but more Sobs, Chao1, and Ace were identified in the TA-treated groups compared with the control groups. By contrast, TA application led to an increase in Sobs, Chao1, and Shannon, as well as an increase in OTUs in stylo silage. This indicates that the bacterial diversity varied considerably, which may have been caused by the forage varieties, silage additives, and fermentation periods. As our previous studies demonstrated, the bacterial diversity of *Neolamarckia cadamba* leaf silage was far lower than for Italian ryegrass (*Festuca perennis*, formerly *Lolium multiflorum*) silage and *Moringa oleifera* leaf silage (He et al., 2019a; Wang et al., 2019a; Yan et al., 2019).

In order to further understand the variance in bacterial communities with TA additives, PCA was employed. Moreover, a clear separation was generated between the control groups and the TA-treated groups for both mulberry leaf and stylo silage, which showed that TA additives had a significant effect on the bacterial communities in these two types of silage. Furthermore, a greater extent of separation was formed in stylo silage than in mulberry leaf silage. As the variations in microbial communities might explain the differences in silage quality, adjusting the concentration and/or quantity of TA additive might help to promote the various microbial communities to achieve a better quality of fermentation.

### Bacterial Abundance of Both Mulberry Leaf and Stylo Silages

*Lachnoclostridium*, a group of gram-positive, motile, and obligately anaerobic spore-forming clostridia within the family *Lachnospiraceae*, can normally grow in mesophilic or thermophilic conditions (20–63°C) and neutral or alkaline pH (7.0–11.0). Strains can also ferment some mono- and disaccharides to produce acetate as the end product. However, oxidase and catalase are not produced in the fermentation process (Yutin and Galperin, 2013). In our study, the relative abundance of *Lachnoclostridium* in mulberry leaf silage was high, which might explain the considerable loss of DM and the high AA and NH_3_-N contents. As He et al. (2019b) reported, *Lachnoclostridium* might function like *Enterobacter* or *Clostridium* spp. according to their phyletic classification in the ensiling process. Furthermore, this unsatisfactory fermentation quality of mulberry leaves was not promoted by TA addition, which might have been because the TA additive had little effect on the relative abundance of *Lachnoclostridium*.

*Kosakonia* is a new genus of aerobic Gram-negative bacteria belonging to the family *Enterobacteriaceae*. In previous studies, *Kosakonia* spp. were isolated mainly from bean and soybean plants (Almeida Lopes et al., 2016), and were also separated from alfalfa root nodules (Fatemeh et al., 2018). As reported, *Kosakonia* had the ability to produce plant growth-promoting properties such as fixing nitrogen and producing indole acetic acid (IAA) deaminase (Fatemeh et al., 2018), indicating a positive effect on nitrogen distribution by reducing molecular nitrogen to NH_3_ and then synthesizing mainly protein. *Klebsiella* spp. are considered to be harmful microorganisms in silage due to their reducing the aerobic stability of fodder (Zhang et al., 2009) and inhibiting growth at pH values below 4.0 (Junges et al., 2017). *Weissella* spp. are considered to be early colonizers as they are inhibited by the decrease in pH as fermentation progresses (Graf et al., 2016). Because of the relatively high pH value of mulberry leaf silage, *Klebsiella* and *Weissella* were detected in the experiments carried out in this study. Most *Weissella* species are obligate heterofermentative bacteria that convert WSC to lactate and acetate as major end products (Graf et al., 2016). Moreover, *Weissella*, *Lactobacillus*, and *Enterococcus* are considered to be the main lactate-producing bacteria present during the ensiling process (Ni et al., 2018). The higher relative abundances of these genera would be expected to produce more acidic conditions and reduce the pH more rapidly. From these observations, these genera were present, although their nondominance might partly explain the fairly high pH of both the mulberry leaf and the stylo silage.

*Clostridium* spp. are Gram-positive, obligate anaerobic, spore-forming bacteria that grow in conditions of relatively high pH (>4.5) and high forage moisture content (>70%; Driehuis et al., 2018). Therefore, they are rapidly inhibited in silage if the pH value falls to 4 or below (Muck, 2010). Generally, *Clostridium* spp. are the most undesirable species that can occur in silage, as they are confirmed to be responsible for excessive protein degradation, DM loss, and even butyric acid production, preventing a rapid fall in pH and permitting the growth of less acid-tolerant spoilage microorganisms (He et al., 2019b; Wang et al., 2019b). Moreover, many *Clostridium* spp. in silage can be hazardous to animal health and the safety of milk and other animal food products (Driehuis et al., 2018). Pathogenic *Clostridium* spp., such as *C. botulinum*, produces the neurotoxin botulinum, which can result in botulism (Schantz and Johnson, 1992), a rare and potentially fatal illness, with a fatality rate of 5–10% in humans and 15–20% in cattle. In our study, the relative abundance of *Clostridium* spp. in stylo silage was 82.8%, which decreased to 3.2–3.8% in silage to which TA had been added. This was consistent with the decrease in DM loss, NH_3_-N, and butyric acid content and also with the increase in TP content. The improvement in silage quality was likely caused by the strong antimicrobial activity of the tannins present in the silage, which indicates that TA additive might be a positive strategy for inhibiting clostridia and promote fermentation quality in high-moisture silage.

*Acinetobacter* spp. have been detected in many types of silage, including in *Moringa oleifera* leaf (Wang et al., 2019b), corn (Nishino et al., 2011), and stylo (He et al., 2019b) silage. Previous studies have shown that *Acinetobacter* spp. are aerobic bacteria, but can also grow in the anaerobic ensiling process by using acetate as a substrate (Fuhs and Chen, 1975). Therefore, as a growth substrate, the greater acetic acid content might explain the greater relative abundance of *Acinetobacter* (6.32%) existed in 2% TA-treated group of stylo silage. This result is similar to that obtained by Ogunade et al. (2018), who found a higher abundance of *Acinetobacter* spp. in LAB-treated silage than in CK silage, suggesting that the increased *Acinetobacter* abundance might result in an improvement in acetic acid content. *Methylobacterium* spp. are considered mainly as a kind of rhizobial branch belonging to the alpha-2 subclass of the *Proteobacteria* (Sy et al., 2001). They are widespread in artificial environments and also occur in natural environments. In addition, *Methylobacterium* has also been detected in table olive fermentation and silage fermentation (He et al., 2019b; Wang et al., 2019b). *Methylobacterium* bacteria are very important in the environmental carbon cycle due to their ability to metabolize various plant decomposition compounds (Holland, 1997). As Madhaiyan et al. (2006) reported, *Methylobacterium* can promote plant growth by producing phytohormones and stimulating germination. However, the specific function of *Methylobacterium* in the fermentation process is still unclear. *Pantoea* spp. has also not been extensively investigated in different types of silage. Ogunade et al. (2018) reported that the relative abundance of *Pantoea* spp. correlated negatively with pH and NH_3_-N in alfalfa silage, which indicates that *Pantoea* may also contribute to protein preservation. The high relative abundance of *Pantoea* spp. in the TA-treated group of stylo silage was concomitant with better fermentation quality and nitrogen preservation. Accordingly, further studies of *Pantoea* spp. should focus on examining different species for their potential roles in fermentation and TP preservation in forage silage because some of them may be good candidates for development into new silage inoculants.

## Conclusion

This study revealed that TA significantly improved the fermentation quality and antioxidant capacity of both mulberry leaf and stylo silage. It also inhibited proteolysis in high-moisture stylo silage. Moreover, the addition of TA increased the relative abundance of *Weissella*, *Acinetobacter*, and *Kosakonia* spp. and inhibited the growth of undesirable *Clostridium* spp. in both mulberry leaf and stylo silage. And the addition of TA showed a more positive effect on stylo silage. It is suggested that TA can be used to improve the quality of silage fermentation of mulberry leaves and stylo and that 2% TA was the better concentration of additive to use.

## Data Availability Statement

Publicly available datasets were analyzed in this study. This data can be found here: the sequences were deposed in the Sequence Read Archive (SRA) with an accession number SRP220981.

## Author Contributions

QZ formulated and designed the experiments. CW, RP, and HL performed the experiments. CW was mainly responsible for analyzing the data and writing the manuscript. WZ and XC were involved in the revision of the manuscript. All authors contributed to the article and approved the submitted version.

### Conflict of Interest

The authors declare that the research was conducted in the absence of any commercial or financial relationships that could be construed as a potential conflict of interest.

## Footnotes

^1^https://qiime.org

^2^http://www.omicshare.com/tools
